# Trade-off strategies between growth and defense of spring ephemeral plants in early spring

**DOI:** 10.3389/fpls.2025.1503169

**Published:** 2025-01-28

**Authors:** Liben Pan, Tianqi Wang, Vladimir L. Gavrikov, Xiaorui Guo, Liqiang Mu, Zhonghua Tang

**Affiliations:** ^1^ School of Forestry, Northeast Forestry University, Harbin, China; ^2^ Key Laboratory of Forest Plant Ecology, Ministry of Education, Northeast Forestry University, Harbin, China; ^3^ College of Chemistry, Chemical Engineering and Resource Utilization, Northeast Forestry University, Harbin, China; ^4^ School Ecology and Geography, Siberian Federal University, Krasnoyarsk, Russia; ^5^ Northeast Asia Biodiversity Research Center, Northeast Forestry University, Harbin, China

**Keywords:** plant functional traits, spring ephemeral plants, plant traits networks, adaptation strategy, elevation

## Abstract

**Introduction:**

Spring ephemeral plants represent a unique ecological category of herbaceous plants, characterized by early blooming and vivid flowers with significant ornamental value. Understanding the adaptive strategies of spring ephemerals is crucial for the introduction and cultivation of early spring plants, as well as for optimizing light energy utilization and nutrient cycling within ecosystems.

**Methods:**

We evaluated 26 functional traits across four spring ephemerals and four spring non-ephemeral plants along an elevation gradient. By establishing a plant functional trait network, we examined the adaptation strategies of early spring plants at different elevations and compared the differences in adaptation strategies between two types of plants.

**Results:**

Spring ephemerals exhibited higher concentrations of carbon and nitrogen, lower concentrations of carbohydrates, higher edge density and modularity in trait networks, and stronger linkages between defense traits. Plants at higher elevations demonstrated higher leaf dry matter content and leaf total flavonoid concentration, and lower nitrogen concentration, influenced by temperature, precipitation, and soil nutrients.

**Discussion:**

These results demonstrated that spring ephemerals have a strong nutrient uptake capacity, and adopt resource competition strategies to rapidly accumulate nutrients and reproduce. The plants at higher elevations adopt more conservative strategies, with trait networks showing increased modularity, edge density, and closer correlations among traits to enhance resource utilization. This study provides new insights into the adaptive strategies of spring ephemerals by demonstrating how plants allocate resources for growth and defense through the regulation of trait variation and correlations among traits.

## 1 Introduction

Spring ephemerals, which primarily inhabit the understory of cold temperate deciduous forests, grow rapidly post-snowmelt and flower and fruit before canopy closure in early summer, culminating into dormancy (Lapointe, 2008). Previous studies often used the phenological changes of spring ephemerals, such as emergence and flowering, as indicators of climate change effects on spring phenology (Hereford et al., 2017; Bucher and Römermann, 2021). Moreover, Spring ephemerals bloom early and have bright flowers that can be used as ornamental plants to fill garden gaps from March to May. As an early stage of dynamic changes in the herbaceous layer, spring ephemerals enhance the efficiency of light energy utilization, reduce soil nutrient loss caused by snowmelt runoff, and improve soil fertility (Alecrim et al., 2023). Plants ecological functions are closely linked to their functional traits (Brown and Anand, 2022). The reconciliation of organ traits provides critical understanding of plant adaptations to dynamic environmental conditions (Rao et al., 2023). Therefore, significant focus should be placed on the response and adaptation strategies of spring ephemerals functional traits to environmental changes for plant conservation and introduction to cultivation.

Plant functional traits reflect the ability of plants to acquire and utilize resources, which is pivotal for understanding plant environmental adaptation strategies (Yang et al., 2019). To date, many important ecological physiology theories have been used to reveal the differences in resource acquisition and environmental adaptation of plants by summarizing crucial physiological traits in different life forms (Schnitzer and Bongers, 2011). For instance, the leaf economic spectrum shows that plants with high leaf nitrogen content, high photosynthetic rates, and short lifespans adopt a quick investment-return strategy by quantifying the relationships between traits such as leaf chemical traits (nitrogen and phosphorus), structural traits (specific leaf weight), physiological traits (photosynthetic capacity and dark respiration rate) and leaf lifespan (Alecrim et al., 2023). Plants with high photosynthetic rates and high leaf nitrogen content usually have higher demand for nutrients but exhibit reduced tolerance to unfavorable environments, such as drought and soil nutrient deficiencies (Zhan et al., 2019; Alecrim et al., 2023). Moreover, plant ecological stoichiometry reveals the link between stoichiometric characteristics and plant growth functions along with environmental factors by investigating the elemental contents and compositions of plants and their variations with environmental factors. For example, the growth rate hypothesis suggests that with increasing growth rate, plant N/P and C/P ratios tend to decrease, while P content tends to increase (Isanta-Navarro et al., 2022). The productivity-leaf longevity hypothesis indicates that leaf nutrients in ephemeral plants influence vegetation productivity during brief growing seasons, thus the relationship between vegetation productivity and leaf nutrient content is higher in ephemeral plants (Tang et al., 2018). Previous studies have consistently demonstrated that the occurrence and development of plant functional traits are significantly influenced by environmental factors. Variations in plant functional traits reflect long-term adaptation of plants to environmental heterogeneity. Furthermore, plant functional traits are also significantly influenced by species evolution (Liu et al., 2024). Adler et al. (2014) linked the global trait database with the empirical matrix population models of 222 species, highlighting a strong relationship between functional traits and plant life history.

Differences in photosynthesis and metabolic traits between spring ephemeral plants and other perennial herbaceous plants have been reported. Spring ephemeral plants have a higher net photosynthetic rate and light compensation point than non-ephemeral plants (Popovic et al., 2016). Additionally, spring ephemeral plants can flower earlier than spring non-ephemeral plants due to their rapid completion of vernalization. This process involves the consumption of energy sources like sugars and amino acids (Yan et al., 2022). As canopy foliage unfolds, resources accumulated in the leaves and stems of spring ephemerals are rapidly transferred to seeds (Houle, 2002). This shows that there are synergies and trade-offs in traits at different stages of plant growth. Therefore, focusing on the linkages among multiple traits in spring ephemeral plants can help understand plant adaptation strategies and their ecosystem functions.

The plant trait network (PTN) and its parameters efficiently quantify multifaceted trait relationships, enhancing the understanding of plant adaptation strategies. Higher degree traits in individual parameters efficiently promote resource utilization and acquisition among plant tissues (He et al., 2020). Kleyer et al. (2019) found that key traits in the PTN of perennial herbaceous plants are biomass allocation traits and stem specific length. In the overall parameters, different plant traits form specific functional modules performing distinct functions. PTNs with higher modularity adapt better to changing environments. High edge density and shorter average path length facilitate efficient resource acquisition and mobilization, promoting trait coordination (Li et al., 2021).

Generally, limited plant resources cause trade-offs between growth maintenance, reproduction, and defense (Bucher and Römermann, 2021). The effects of growth maintenance and resource allocation on survival are major determinants of adaptive strategies in plants with different life histories (Gast et al., 2020). Elevation is a major factor influencing variation in plant functional traits, making an ideal place for studying plant trade-offs between growth and defense. We hypothesized that spring ephemeral plants adopt a quick investment-return strategy, exhibiting higher modularity of functional trait networks and stronger adaptability to adverse environments. To analyze this, we assessed 26 functional traits, including 7 economic traits, 11 nutrient traits, and 8 defensive traits, in 4 spring non-ephemeral plants and 4 spring ephemeral plants at different elevations. (1) How do differences in functional traits and trait networks between spring ephemeral and non-ephemeral plants reflect their ecological adaptation strategies? (2) Are these trait changes influenced by phylogenetic constraints? (3) How does the elevational gradient drive the variation in plant functional traits by influencing environmental factors?

## 2 Materials and methods

### 2.1 Experimental design and plant materials

This study site is located on Sifang Mountain, Jilin Province, China. The climate is East Asian monsoon climate, with a mean annual temperature of 5.5°C and mean total annual precipitation of 880 mm. The elevation range of the study area spans from 610 to 1283 m. Field investigations indicate that as elevation increases, the diversity of herbaceous plants in the understory declines, and the plant species become more uniform. Broadleaf secondary forests represent the typical forest vegetation of this region. The dominant tree species in the canopy layer include *Quercus mongolica* Fisch. ex Ledeb., *Juglans mandshurica* Maxim., and *Larix gmelinii* (Rupr.) Kuzen.; the main dominant species in the shrub layer are *Sorbaria sorbifolia* (L.) A. Braun, *Syringa reticulata* subsp. amurensis (Rupr.) P. S. Green & M. C. Chang and *Lonicera japonica* Thunb., among others. Therefore, three species-rich elevation ranges were selected: 620.79–625.50 m, 827.60–834.94 m and 1020.37–1022.74 m. The slope, latitude, longitude, and elevation of each sampling site were recorded using a clinometer and geographic positioning system (GPS), respectively. Environmental factors at different elevations, based on the longitude and latitude of the sample sites, were collected from WorldClim (https://www.worldclim.org/). **Table 1** provides a detailed description of the climate and soil physicochemical characteristics of the sampling sites.

**Table 1:** Soil physicochemical characteristics and climate of sample sites at different elevation.

	600	800	1000
soil_pH	6.187 ± 0.101a	5.77 ± 0.093a	5.617 ± 0.267a
soil_C (%)	6.84 ± 0.261c	8.877 ± 0.299b	11.243 ± 0.09a
soil_P (%)	0.1 ± 0a	0.08 ± 0.006a	0.083 ± 0.009a
soil_N (%)	1.073 ± 0.009a	0.803 ± 0.009c	0.953 ± 0.018b
soil_EC (μs/cm)	176 ± 42.143a	132 ± 10.149a	129.333 ± 30.118a
MAP (mm)	69.23 ± 0.046c	71.593 ± 0.043b	72.6 ± 0.321a
MASR (kJ m^-2^ day^-1^)	13715.83 ± 0a	13559.75 ± 0b	13559.113 ± 13.057b
MAT (°C)	3.82 ± 0a	2.98 ± 0b	2.507 ± 0.193c

MAT is mean annual temperature. MAP is mean annual precipitation. MASR is mean annual solar radiation. Different lower-case letters indicate a significant difference (P< 0.05) as determined by one-way ANOVA.

In May 2022, a survey of herbaceous plants was conducted at different elevations on Sifang Mountain. At each elevation, three 10 m×10 m plots were established. Within each plot, five 1 m×1 m quadrats were placed at the center and four corners. The species, number, height, and cover of all herbaceous plants in each sample plot were measured and recorded. The important value (IV) of each species at different elevations was calculated as follows:


IV=(relative frequency+relative density+relative coverage)/3

Herb species with the highest importance values, ranked in the top 15 at each elevation, are listed in **Supplementary Table S1**. Eight plants common to each elevation were selected and categorized into two groups based on their phenology. One group consists of spring ephemeral plants, which bloom from March to May and become dormant in early summer, with a short aboveground growth cycle (Heberling et al., 2019), including *Anemone raddeana* Regel, *Erythronium japonicum* Decne., *Adonis amurensis* Regel & Radde and *Hylomecon japonicum* (Thunb.) Prantl (**Supplementary Figure S1**). As a control, another group is defined as spring non-ephemeral plants, emerging in April, and extending growth into early fall, with a long aboveground growth cycle, including *Angelica dahurica* (Fisch. ex Hoffm.) Benth. & Hook. f. ex Franch. & Sav., *Aegopodium alpestre* Ledeb., *Cardamine leucantha* (Tausch) O. E. Schulz and *Pimpinella brachycarpa* (Kom.) Nakai. The details for each species are listed in **Supplementary Table S2**.

At least 5 plants of similar size with fully expanded leaves were collected for each species in 15 randomized plots at each sampling site. A total of 26 functional traits were determined and classified into 3 categories: economic, nutrient, and defensive traits (Li et al., 2022; Mohanbabu et al., 2023). Economic traits included leaf dry matter content (LDMC), specific leaf area (SLA), leaf carbon concentration (LC), leaf nitrogen concentration (LN), leaf phosphorus concentration (LP), the ratio of leaf carbon to nitrogen concentration (LC/N), and the ratio of leaf nitrogen to phosphorus concentration (LN/P). Nutrient traits included root carbon concentration (RC), root nitrogen concentration (RN), root phosphorus concentration (RP), the ratio of root carbon to nitrogen concentration (RC/N), the ratio of root nitrogen to phosphorus concentration (RN/P), leaf soluble sugar concentration (LSS), leaf starch concentration (LS), leaf nonstructural carbohydrates (LNSC), root soluble sugar concentration (RSS), root starch concentration (RS) and root nonstructural carbohydrates (RNSC). Defensive traits included leaf cellulose content (LCC), leaf lignin content (LLC), leaf total phenols concentration (LTPC), leaf total flavonoids concentration (LTFC), root cellulose content (RCC), root lignin content (RLC), root total phenols concentration (RTPC), and root total flavonoids concentration (RTFC). The classification, abbreviations and units of all functional traits are listed in **Supplementary Table S3**.

### 2.2 Measurement of functional traits

Unfolded leaves were scanned to determine the leaf area. Fresh leaves were dried at 60°C, and pre- and post-drying masses recorded separately. The ratio of leaf dry weight to leaf fresh weight defines LDMC. SLA is the ratio of leaf area to dry weight.

Plant leaf and root samples were dried, ground, and passed through a 0.5 mm fine sieve. The carbon and nitrogen concentrations were measured using an elemental analyzer (Vario MACRO Cube, Elementar, Germany). The phosphorus concentration was determined using the vanadium molybdate blue colorimetric method (Wieczorek et al., 2022). The anthrone method was used to determine the concentrations of soluble sugars and starch (Mesa et al., 2016). Nonstructural carbohydrates are the sum of soluble sugars and starch (Du et al., 2020). The Folin-Ciocalteu method was used to determine the total phenol concentration (Abramovic et al., 2018). Total flavonoid concentration was determined using the aluminum nitrate-sodium nitrite colorimetric method (Sakanaka et al., 2005). Cellulose content was determined using the sulfuric acid anthrone method (Dampanaboina et al., 2021). The lignin content was determined using the acetylation method, which causes the phenolic hydroxyl groups in lignin to undergo acetylation (Fukushima and Hatfield, 2004). Additional measurement details of the plant traits are presented in **Appendix S1**.

### 2.3 Statistical analysis

The Shapiro-Wilk test was used to assess the normality of the data. Traits (RCC, LCC, LC), which were normally distributed, were compared between spring ephemeral and non-ephemeral plants using the paired t-test. For the remaining 23 traits that did not follow a normal distribution, the Mann-Whitney test was applied to assess differences between the two groups. All trait data were standardized, and principal component analysis (PCA) was performed to test for interspecific differences in functional traits.

To quantify the variation patterns of functional traits along elevation and between plant types (spring ephemeral and non-ephemeral plants), we established separate linear models for each trait. Species, elevation, and the interaction between species and elevation were used as fixed effects. Linear regression analyses were performed to evaluate the relationships between the 26 functional traits of spring ephemeral and non-ephemeral plants with soil physicochemical characteristics and climate of sample sites. Before analysis, traits were transformed to conform to a normal distribution. All statistical analyses were performed using R 4.3.2 software (R Core Team, 2023).

**Figure 1:**
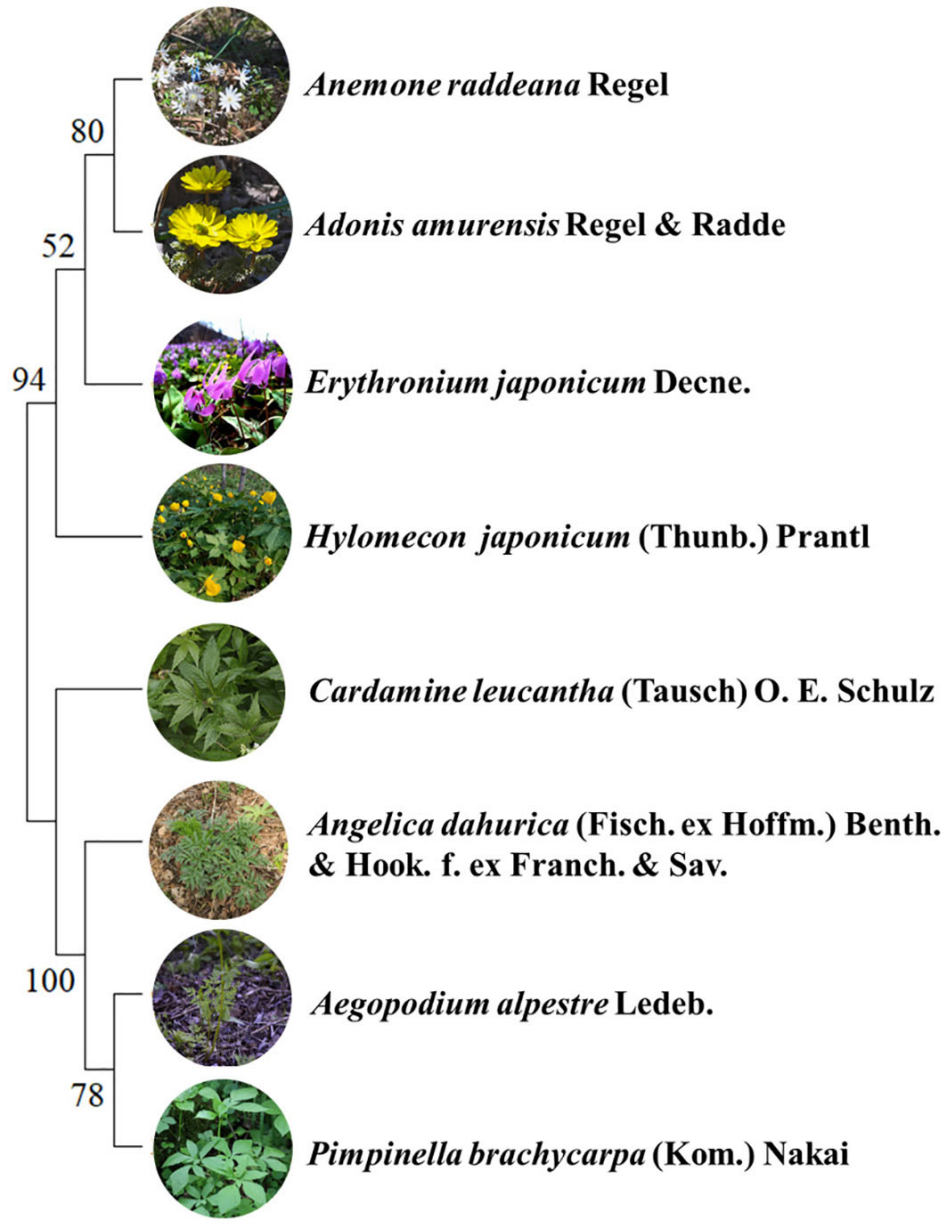
Phylogenetic tree of four spring ephemeral plants and four spring non-ephemeral plants.

To test the influence of phylogeny on functional traits, chloroplast genomes of species from the study area were collected through NCBI database. A phylogenetic tree was constructed in MEGA11 software using the maximum likelihood method (**Figure 1**), with 1000 bootstrap replicates performed to assess node support (Tamura et al., 2021). The phylogenetic signal was analyzed using the *K* test developed by Blomberg et al. (2003) *K*= 1 represents the Brownian motion model of evolution. *K*>1 indicate that functional traits exhibit stronger phylogenetic signals than expected under the Brownian motion model, while *K*<1 suggest weaker phylogenetic signals. The significance of the phylogenetic signal was assessed by comparing the observed *K* with *K* generated under a null model. Null model *K* were generated by randomly shuffling species at the tips of the phylogenetic tree 999 times. If the null model *K* fall below the observed *K* (*P*< 0.05), the functional trait is considered to exhibit significant phylogenetic signals; otherwise, the phylogenetic signal is classified as non-significant. The *K*, calculated using the “picante” package in R software, was used to detect phylogenetic signals among traits.

To compare differences in adaptation strategies between the two plant types and different elevations, plant functional trait networks (PTNs) were constructed. Firstly, the Spearman’s correlation coefficients between traits were calculated. To avoid spurious correlations, relationships with |r| >0.2, *P*< 0.05 were set to 1; otherwise, they were set to 0. A data matrix was constructed such that if the correlation value between two traits was 1, there was an edge connection; if the value was 0, there was no edge connection. Finally, the “iggraph” package in R software was used to visualize the PTNs and calculate network parameters, such as degree, edge density, and modularity. The definitions and ecological significance of the network parameters are listed in **Supplementary Table S4**. Additionally, to compare the importance of different functional traits in the plant functional network, the relative importance of each trait was calculated. Relative importance is defined as the average degree of each type of trait divided by the sum of all trait degrees.

## 3 Results

### 3.1 The variation of plant functional traits between spring ephemeral and non-ephemeral plants

**Figure 2:**
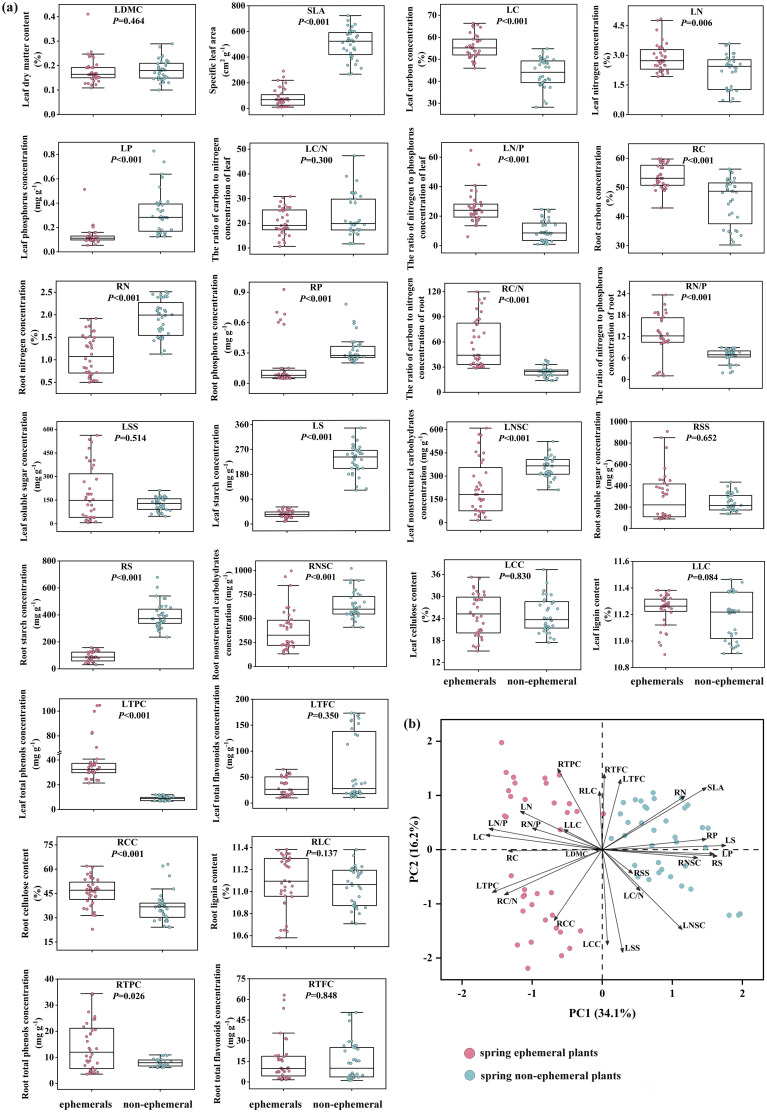
Variation of 26 traits between spring ephemeral plants and spring non-ephemeral plants **(A)**; principal component analysis of 26 functional traits **(B)**. LDMC, leaf dry matter content; SLA, specific leaf area; LC, leaf carbon concentration; LN, leaf nitrogen concentration; LP, leaf phosphorus concentration; LC/N, the ratio of carbon to nitrogen concentration of leaf; LN/P, the ratio of nitrogen to phosphorus concentration of leaf. Nutrient traits include: RC, root carbon concentration; RN, root nitrogen concentration; RP, root phosphorus concentration; RC/N, the ratio of carbon to nitrogen concentration of root; RN/P, the ratio of nitrogen and phosphorus concentration of root; LSS, leaf soluble sugar concentration; LS, leaf starch concentration; LNSC, leaf nonstructural carbohydrates; RSS, root soluble sugar concentration; RS, root starch concentration; RNSC, root nonstructural carbohydrates. Defensive traits include: LCC, leaf cellulose content; LLC, leaf lignin content; LTPC, leaf total phenols concentration; LTFC, leaf total flavonoids concentration; RCC, root cellulose content; RLC, root lignin content; RTPC, root total phenols concentration; RTFC, root total flavonoids concentration.

For all traits, LC, LN, LN/P, RC, RC/N, RN/P, LTPC, RTPC, and RCC were significantly lower in spring non-ephemeral plants than in spring ephemerals (*P*< 0.05), while spring non-ephemeral plants exhibited higher SLA, LP, RN, RP, LS, LNSC, RS, and RNSC (**Figure 2A**). In economic traits, spring ephemeral plants showed higher LC, LN, and LN/P, while spring non-ephemeral plants showed higher SLA and LP. In nutrient traits, LP, RN, RP, LS, LNSC, RS, and RNSC in spring non-ephemeral plants were significantly higher than spring ephemeral plants (*P*<0.05). Notably, most defensive traits of spring ephemeral plants were higher than those of spring non-ephemeral plants, with LTPC, RTPC, and RCC showing significant increases in spring ephemerals (*P*<0.05).

PCA of plant traits suggested significant differentiation between spring ephemeral and non-ephemeral plants on the PC1 axis (**Figure 2B**). PC1 was closely associated with the variation in LS, LC, LP, LN/P, and RS, explained 34.1% of the total variance (**Figure 2B**; **Supplementary Table S5**). Variations in LSS, LCC, RTPC, LNSC, and RTFC along the PC2 axis also differed significantly between the two plnant types (**Figure 2B**), explaining 16.2% of the total variance.

### 3.2 The variation of plant functional traits along elevation gradients

**Figure 3:**
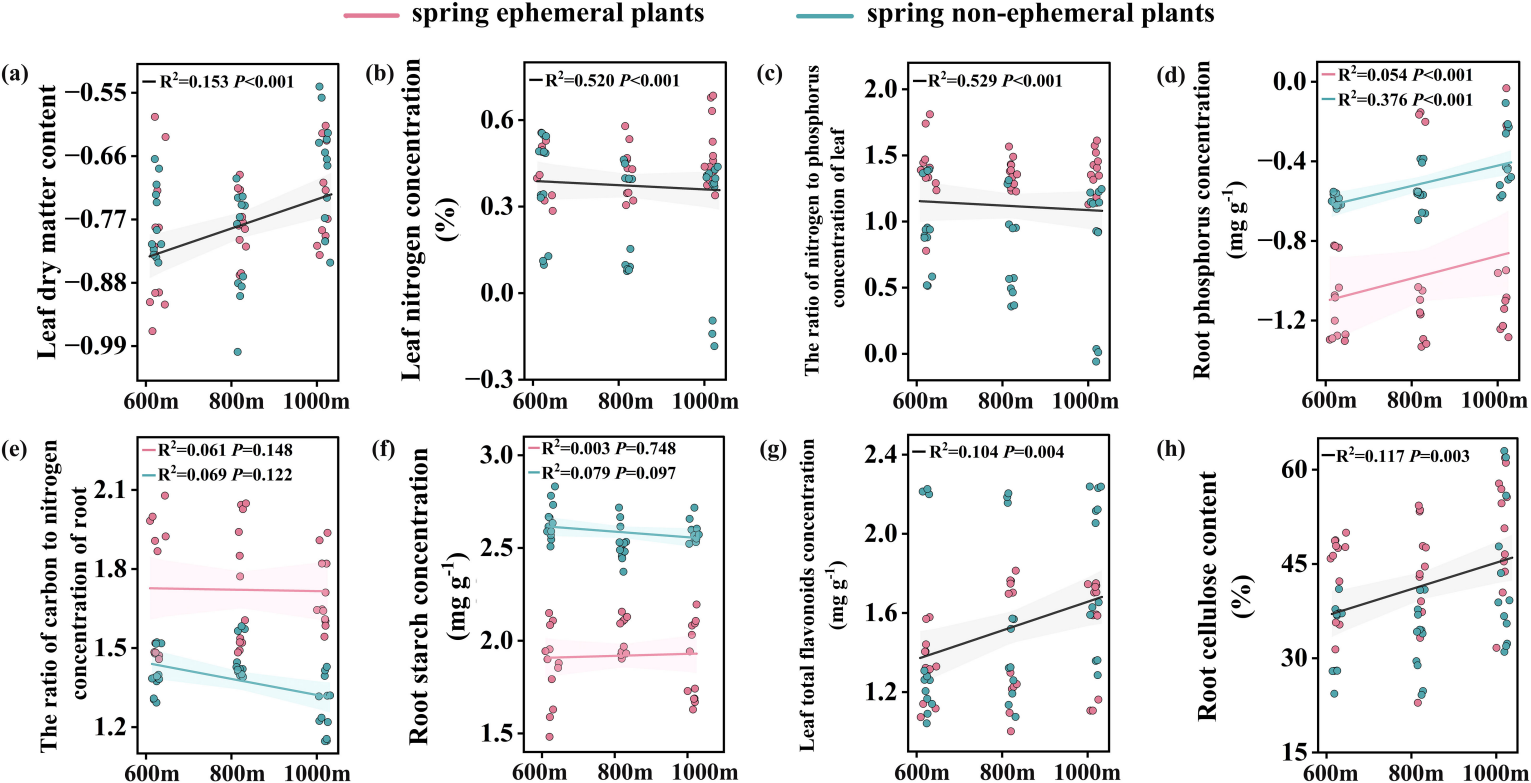
Effect of elevation on LDMC, LN, LNP, RP, RCN, RS, LTFC, RCC **(A-H)** of plants with different species. When the statistical model detected the interaction between species and elevation, the changes along elevation gradient between the two types of plants were constructed separately. The black line indicated a significant (P< 0.05) elevation effect for the trait (no interaction). Spring ephemeral plants were represented by pink and spring non-ephemeral plants were represented by blue. The shaded area around the line represents the 95% confidence interval of the regression. LDMC, leaf dry matter content; LN, leaf nitrogen concentration; LN/P, the ratio of nitrogen to phosphorus concentration of leaf; RP, root phosphorus concentration; RC/N, the ratio of carbon to nitrogen concentration of root; RS, root starch concentration; LTFC, leaf total flavonoids concentration; RCC, root cellulose content.

We detected significant effects of elevation and species, but not their interaction, on LN, LN/P, and RCC (**Table 2**). These traits differed across plant species, but their variations with elevation were similar (**Figures 3B, C, H**). RCC increased by 21.19% at higher elevations compared to lower elevations. Additionally, LDMC and LTFC were significantly affected by elevation, with LDMC increasing on average 1.27-fold and LTFC increasing 1.72-fold relative to the lowest elevation (**Figures 3A, G**). Plant types and elevation displayed significant main and interactive effects on RP, RC/N, and RS (**Table 2**), suggesting that the magnitude of change in these variables with elevation varied among species. Therefore, we constructed models for different species, using elevation as a fixed factor (**Figures 3D–F**). The results showed that only RP increased significantly along the altitudinal gradient for both plant types. No main effect or interaction effect of elevation was found on the other 18 traits (**Table 2**).

**Table 2:** Statistics for linear models of 26 functional trait values along elevation, incorporating species, elevation, and their interaction as fixed effects.

Variables	Effect					
	Species		Elevation		Species * Elevation	
	F	*P*	F	*P*	F	*P*
Leaf dry matter content (LDMC)	0.307	0.582	9.032	**0.000**	0.941	0.395
Specific leaf area (SLA)	152.604	**0.000**	0.493	0.613	0.651	0.525
Leaf carbon concentration (LC)	74.536	**0.000**	2.243	0.114	1.23	0.299
Leaf nitrogen concentration (LN)	16.117	**0.000**	5.035	**0.009**	2.629	0.080
Leaf phosphorus concentration (LP)	34.654	**0.000**	0.429	0.653	0.102	0.903
The ratio of carbon to nitrogen concentration of leaf (LC/N)	2.300	0.134	2.786	0.069	2.123	0.128
The ratio of nitrogen to phosphorus concentration of leaf (LN/P)	62.095	**0.000**	7.128	**0.002**	1.979	0.146
Root carbon concentration (RC)	30.286	**0.000**	1.003	0.372	2.156	0.124
Root nitrogen concentration (RN)	68.258	**0.000**	2.000	0.143	1.830	0.169
Root phosphorus concentration (RP)	10.922	**0.002**	0.208	0.813	3.156	**0.049**
The ratio of carbon to nitrogen concentration of root (RC/N)	54.647	**0.000**	2.600	0.082	3.708	**0.030**
The ratio of nitrogen and phosphorus concentration of root (RN/P)	31.668	**0.000**	1.605	0.209	2.399	0.099
Leaf soluble sugar concentration (LSS)	0.070	0.792	0.586	0.560	1.724	0.186
Leaf starch concentration (LS)	661.982	**0.000**	5.002	0.109	0.681	0.510
Leaf nonstructural carbohydrates (LNSC)	25.234	**0.000**	1.257	0.291	1.001	0.373
Root soluble sugar concentration (RSS)	0.048	0.827	0.17	0.844	0.760	0.472
Root starch concentration (RS)	398.065	**0.000**	0.371	0.692	5.581	**0.006**
Root nonstructural carbohydrates (RNSC)	42.195	**0.000**	0.057	0.945	2.283	0.110
Leaf cellulose concentration (LCC)	0.046	0.831	1.127	0.33	0.744	0.479
Leaf lignin content (LLC)	2.487	0.120	1.284	0.284	0.175	0.840
Leaf total phenols concentration (LTPC)	347.659	**0.000**	1.166	0.318	2.030	0.139
Leaf total flavonoids concentration (LTFC)	3.744	0.057	4.295	**0.018**	0.572	0.567
Root cellulose concentration (RCC)	23.204	**0.000**	8.253	**0.001**	0.255	0.776
Root lignin content (RLC)	0.400	0.529	1.611	0.207	0.164	0.849
Root total phenols concentration (RTPC)	8.904	**0.004**	1.177	0.315	0.089	0.915
Root total flavonoids concentration (RTFC)	0.037	0.848	0.996	0.375	0.183	0.833

Bolded text indicates that the trait was significantly affected by elevation, species, or interaction. * represents the interaction between variables.

Linear regression analysis was conducted to evaluate the effects of climate factors and soil physicochemical properties along elevation on plant traits. The results revealed that the majority of traits in non-ephemeral plants were significantly influenced by environmental variables (**Supplementary Table S6**). Increases in MAT notably enhanced specific SLA and RN/P, while reducing RCC (**Supplementary Figure S2**), indicating that temperature fluctuations play a crucial role in regulating plant resource allocation. Similarly, higher soil organic carbon content was associated with reduced SLA and RTPC, alongside increased accumulation of leaf secondary metabolites such as total phenols and flavonoids (**Supplementary Figure S3**). Additionally, greater soil nitrogen content significantly decreased the LC/N and promoted higher concentrations of RNSC and RCC (**Supplementary Figure S4**).

### 3.3 The relationship between functional traits and phylogeny

Using the method proposed by Blomberg et al., the phylogenetic signal (*K*) of 26 functional traits across 8 dominant herbaceous plant was analyzed. The 25 traits did not exhibit significant phylogenetic conservatism (*K*<1) and were more strongly influenced by habitat during evolution (**Table 3**). In contrast, the *K* of LTPC was 1.334 (*P*< 0.05), demonstrating a strong phylogenetic signal and significant genetic influence. This finding suggests that species with smaller phylogenetic distances exhibit greater similarity in LTPC.

**Table 3:** Phylogenetic signal of 26 functional trait.

Functional trait	*K*	*P*
Leaf dry matter content (LDMC)	0.035	0.515
Specific leaf area (SLA)	0.372	0.995
Leaf carbon concentration (LC)	**0.034**	**0.003**
Leaf nitrogen concentration (LN)	**0.076**	**0.015**
Leaf phosphorus concentration (LP)	**0.277**	**0.002**
The ratio of carbon to nitrogen concentration of leaf (LC/N)	0.041	0.840
The ratio of nitrogen to phosphorus concentration of leaf (LN/P)	**0.584**	**0.007**
Root carbon concentration (RC)	**0.013**	**0.009**
Root nitrogen concentration (RN)	0.098	0.991
Root phosphorus concentration (RP)	0.193	0.961
The ratio of carbon to nitrogen concentration of root (RC/N)	**0.397**	**0.005**
The ratio of nitrogen and phosphorus concentration of root (RN/P)	**0.290**	**0.019**
Leaf soluble sugar concentration (LSS)	0.890	0.349
Leaf starch concentration (LS)	0.366	0.992
Leaf nonstructural carbohydrates (LNSC)	0.242	0.741
Root soluble sugar concentration (RSS)	0.501	0.302
Root starch concentration (RS)	**0.310**	**0.015**
Root nonstructural carbohydrates (RNSC)	0.169	0.925
Leaf cellulose concentration (LCC)	0.038	0.359
Leaf lignin content (LLC)	**0.001**	**0.000**
Leaf total phenols concentration (LTPC)	**1.334**	**0.044**
Leaf total flavonoids concentration (LTFC)	0.746	0.658
Root cellulose concentration (RCC)	**0.057**	**0.044**
Root lignin content (RLC)	0.001	0.484
Root total phenols concentration (RTPC)	0.726	0.188
Root total flavonoids concentration (RTFC)	0.830	0.611

Note:The K with statistical significance (P < 0.05) are highlighted in bold.

### 3.4 Plant trait networks variation of different plant species

**Figure 4:**
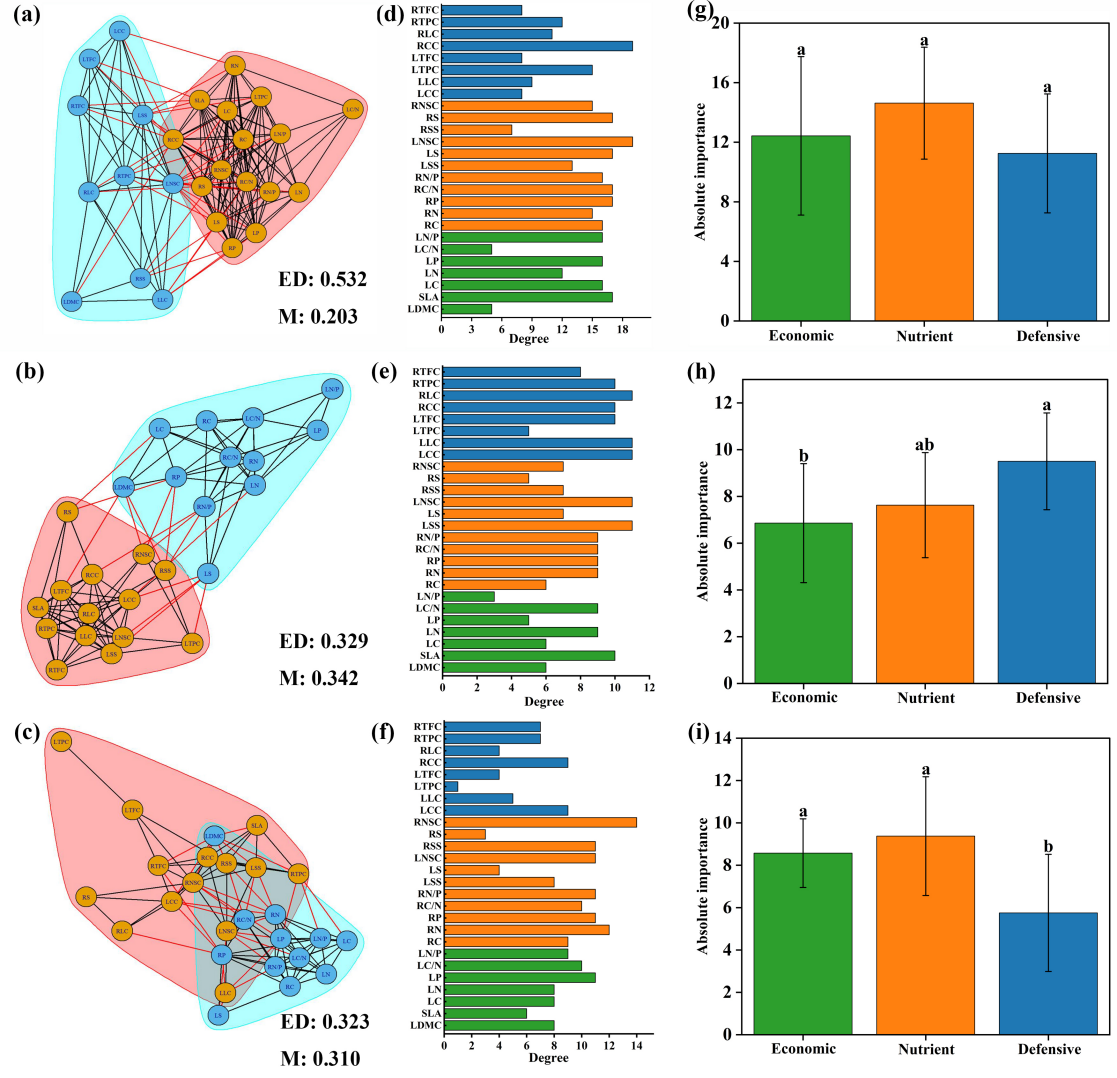
Trait networks of 26 plant traits for all species **(A)**, spring ephemeral plants **(B)** and spring non-ephemeral plants **(C)**. ED indicates edge density. M indicates modularity. Traits with consistent background colors were considered to belong to the same module. The degree of 26 plant traits for all species **(D)**, spring ephemeral plants **(E)** and spring non-ephemeral plants **(F)**. Economic, nutrient and defensive traits are represented by green, orange, and blue colors, respectively. Absolute importance of economic, nutrient and defensive traits for all species **(G)**, spring ephemeral plants **(H)** and spring non-ephemeral plants **(I)**. LDMC leaf dry matter content; SLA, specific leaf area; LC, leaf carbon concentration; LN, leaf nitrogen concentration; LP, leaf phosphorus concentration; LC/N, the ratio of carbon to nitrogen concentration of leaf; LN/P, the ratio of nitrogen to phosphorus concentration of leaf; RC, root carbon concentration; RN, root nitrogen concentration; RP, root phosphorus concentration; RC/N, the ratio of carbon to nitrogen concentration of root; RN/P, the ratio of nitrogen and phosphorus concentration of root; LSS, leaf soluble sugar concentration; LS, leaf starch concentration; LNSC, leaf nonstructural carbohydrates; RSS, root soluble sugar concentration; RS, root starch concentration; RNSC, root nonstructural carbohydrates; LCC, leaf cellulose content; LLC, leaf lignin content; LTPC, leaf total phenols concentration; LTFC, leaf total flavonoids concentration; RCC, root cellulose content; RLC, root lignin content; RTPC, root total phenols concentration; RTFC, root total flavonoids concentration.

Different plant trait networks were constructed, and parameters were calculated based on the data from 26 plant traits across all species (**Figure 4**). The average edge density was 0.34, ranging from 0.323 to 0.532, and the modularity averaged 0.22, ranging from 0.159 to 0.342. The degrees of the 26 traits varied across the networks. Nutrient traits were more important than economic and defensive traits in the trait networks across all species, but there were no significant differences between the three categories (**Figure 4G**).

The two types of plant trait parameters differed. The modularity of trait networks in spring ephemerals was higher than that in non-ephemeral plants. The correlations between LSS, LNSC, LCC, LLC, RLC, and other traits in the plant functional trait network of spring ephemeral plants was relatively high (**Figure 4E**). The correlation of the trait network between RNSC and other traits was highest in spring non-ephemeral plants (**Figure 4F**). Defensive traits were the most important in spring ephemeral plants, while the absolute importance of economic and nutrient traits in spring non-ephemeral plants was similar; both were more important than defensive traits (**Figures 4H, I**).

### 3.5 Plant trait networks variation of different elevations

**Figure 5:**
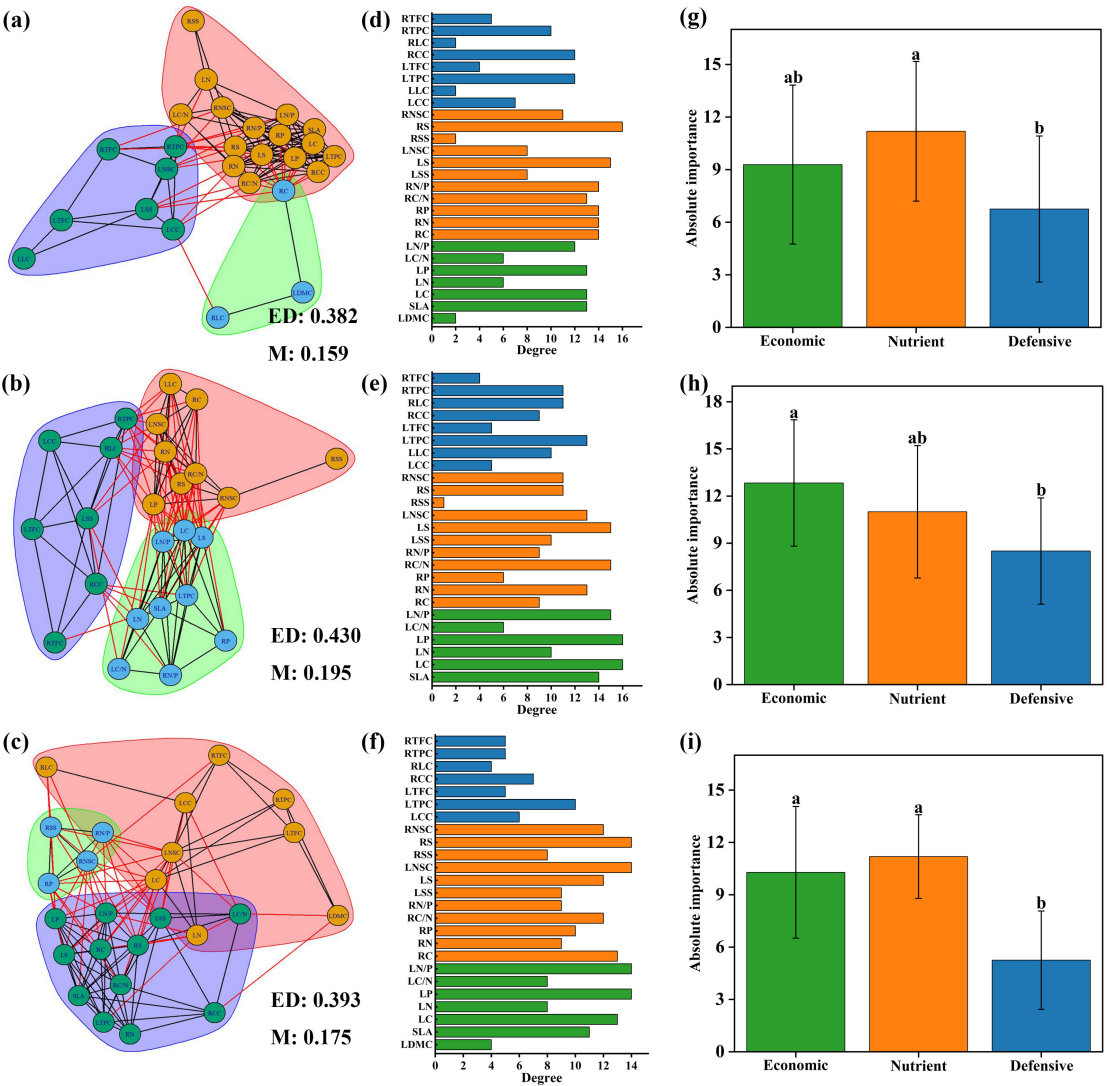
Trait networks of 26 plant traits for low elevation area **(A)**, middle elevation area **(B)** and high elevation area **(C)**. ED indicates edge density; M indicates modularity. Traits with consistent background colors were considered to belong to the same module. The degree of 26 plant traits for low elevation area **(D)**, middle elevation area **(E)** and high elevation area **(F)**. Economic, nutrient and defensive traits are represented by green, orange, and blue colors, respectively. Absolute importance of economic, nutrient and defensive traits for ow elevation area **(G)**, middle elevation area **(H)** and high elevation area **(I)**. LDMC, leaf dry matter content; SLA, specific leaf area; LC, leaf carbon concentration; LN, leaf nitrogen concentration; LP, leaf phosphorus concentration; LC/N, the ratio of carbon to nitrogen concentration of leaf; LN/P, the ratio of nitrogen to phosphorus concentration of leaf; RC, root carbon concentration; RN, root nitrogen concentration; RP, root phosphorus concentration; RC/N, the ratio of carbon to nitrogen concentration of root; RN/P, the ratio of nitrogen and phosphorus concentration of root; LSS, leaf soluble sugar concentration; LS, leaf starch concentration; LNSC, leaf nonstructural carbohydrates; RSS, root soluble sugar concentration; RS, root starch concentration; RNSC, root nonstructural carbohydrates; LCC, leaf cellulose content; LLC, leaf lignin content; LTPC, leaf total phenols concentration; LTFC, leaf total flavonoids concentration; RCC, root cellulose content; RLC, root lignin content; RTPC, root total phenols concentration; RTFC, root total flavonoids concentration.

Plant trait networks were constructed for all species at different elevations (**Figures 5A–C**). Modularity at different elevations had varying trait compositions and was more pronounced at middle and high elevations, accompanied by higher edge density. The nutrient trait RNSC was most strongly linked to other traits among PTNs at low elevations (**Figure 5B**). LC and LP were the most important factors in the trait networks at middle elevations (**Figure 5E**). LN/P, LP, LNSC, and RNSC were the most important factors in the trait networks at high elevation (**Figure 5F**). Compared to defensive traits, the absolute importance of nutrient traits was higher at both low and high elevations, whereas economic traits were higher at middle elevations (**Figures 5G–I**).

## 4 Discussion

The principal component analysis of 26 functional traits clearly reflected the differences between the two types of plants, with PC1 and PC2 contributing 50.3% to group differentiation. From the loading scores of traits, leaf carbon (LC), phosphorus (LP), and starch (LS, RS) concentrations were the most significant in the intergroup differentiation of PC1 (**Figure 2B**; **Supplementary Table S5**). Starch provides energy and carbon skeletons for plants, with root starch serving as a long-term energy reserve for plant dormancy and seed germination (Smith and Zeeman, 2020). Phosphorus plays a crucial role in plant growth and metabolism, regulating sugar and starch production and transport, and impacting the development and maturation of plant roots and seeds (Zhang et al., 2021). These findings emphasize the importance of nutrient accumulation and stoichiometric balance in both types of plants.

Spring ephemeral plants and spring non-ephemeral plants are both understory herbaceous plants that utilize early spring ecological niches, but they exhibit different adaptation strategies to change in the understory light environment. As the canopy foliage expands, light availability in the understory decreases. Spring ephemerals, constrained by light availability, complete their aboveground lifecycle quickly, exhibiting a “shade avoidance” strategy. Conversely, spring non-ephemeral plants extend their lifespan by adjusting their photosynthetic organs, exhibiting a “shade tolerance” strategy (Popovic et al., 2016). Many studies have shown that plant functional traits are significantly influenced by phylogenetic history during their evolutionary processes. However, this study found significant phylogenetic signals in only one of the 26 functional traits, with the others predominantly shaped by environmental factors. In our study, spring non-ephemeral plants showed higher SLA to enhance shade tolerance. Similar findings were reported by Legner et al. (2014), who found that high SLA enhanced photosynthetic yield and helped plants adapt to low-light conditions. These adaptations were reflected in economic traits, nutrient traits, and other physiological and ecological functional traits, resulting in different adaptation strategies for the two types of plants.

The life cycle of herbaceous plants mainly involves growth and reproduction, accompanied by the allocation and storage of energy and adaptation to adverse environments. Our results revealed distinct differences in economic, nutrient, and defensive traits between spring ephemeral and non-ephemeral plants. Spring ephemerals showed higher carbon, nitrogen concentration, N/P ratio, and total phenol concentration in leaves and roots (**Figure 2A**). Changes in nutrient content and stoichiometry affect growth rate and reveal the correlation between nutrient allocation and environmental adaptation. Higher total phenol concentration in spring ephemerals contributes to the antioxidant capacity, thereby enhancing the adaptability to low-temperature environments (Angmo et al., 2021). Carbon accumulation in spring ephemerals was associated with greater photosynthetic capacity. Generally, carbon fixation in plants requires the involvement of numerous proteases (nitrogen pools) and nucleic acids (phosphorus pools) (Mu and Chen, 2021). Nitrogen and phosphorus collaborate in basic metabolic processes in plants, maintaining consistency under environmental pressure (Xiong et al., 2022). However, our study observed nitrogen accumulation only in spring ephemerals, whereas phosphorus accumulation was higher in spring non-ephemeral plants. This may be related to plant genetics or different trade-off strategies for nutrient utilization efficiency during carbon fixation (Dijkstra et al., 2016). Additionally, spring ephemerals have higher C/N ratios in roots, suggesting higher nutrient utilization efficiency. Spring ephemeral plants start growing at low temperatures in early spring and quickly transition to flowering and reproductive growth, leading to higher energy requirements. As a result, LS, LNSC, RS, and RNSC were lower in spring ephemeral plants than in spring non-ephemeral plants. This is consistent with the findings of Popovic et al. (2016), who reported that the growth of spring ephemeral plants is limited by environmental factors and seasonal variations, as well as by the associated sinks for carbohydrate accumulation and nutrient transport. In summary, these traits indicate that spring ephemeral plants have stronger nutrient absorption and utilization abilities, and tend to adopt a fast-growing resource competition strategy.

To better understand the trade-off between plant growth and defense, we studied the variation trends of 26 functional traits across different elevations. In the linear models, LDMC, RP, LTFC, and RCC increased with elevation. High cellulose content enhances the tensile strength of roots. Dantas et al. (2013) showed that plants adopt a conservative strategy under low soil water conditions by increasing LDMC and enhancing the stretching capacity. Plants grow in early spring using water from snowmelt (Sharma et al., 2024), and water utilization is lower at high elevations due to low temperatures and slow snowmelt. Similar results were obtained in our study, where plants showed increased LDMC and root cellulose content at high elevations, indicating that plants tend to adopt resource conservation strategies under resource-limited conditions. However, in non-ephemeral plants, LDMC, RP, LTPC, RTPC, and RCC show significant positive correlations with MAP as elevation and precipitation increase. This contradicts our previous findings, likely due to early spring soil water availability, driven by temperature, differs from annual precipitation patterns along elevation gradients. High LTFC content was detected at high elevations and showed a significant negative correlation with MAT. Flavonoid accumulation helps enhance the antioxidant activity of plants and mitigate damage in harsh environments and against herbivores (Wari et al., 2022). In addition, we observed low leaf nitrogen content at high elevations. Lower nitrogen levels represent low photosynthetic capacity, and higher LDMC represents greater resistance. Thus, an increase in plant defenses at high elevations is accompanied by a decrease in growth costs.

Plant traits with a high degree of importance can be considered pivotal traits, facilitating efficient resource utilization and access. Our study found that NSC in roots is a pivotal trait in spring non-ephemeral plants. In perennial plants, the accumulation of NSC in roots can be used to increase reproductive yield and promote7nbsp;germination in subsequent growth seasons (Watts and Tenhumberg, 2021). In this study, the NSC in roots of spring ephemeral plants were significantly lower than those in spring non-ephemeral plants. This variation may result from their different growth stages, where spring ephemeral plants allocate more carbon sources for reproduction. Compared to late-flowering plants, the most important traits in spring ephemeral plants are LSS, LNSC, LCC, and LLC. Spring ephemeral plants utilize carbohydrates primarily for leaf construction. Yoon et al. (2021) showed that flowering depends on the availability of carbohydrates and the transport of sugars in the phloem. These results indicate that carbohydrates play a crucial role in the growth and flowering stages of spring ephemeral plants, demonstrating their adaptive strategy.

In the plant functional trait network, the modularity of spring ephemeral plants was greater than that of spring non-ephemeral plants, indicating that the spring ephemeral plants are more independent in exercising their functions than spring non-ephemeral plants. Furthermore, highly modular plant trait networks demonstrate enhanced flexibility, suggesting that spring ephemeral plants have a greater adaptive capacity to the environment (Flores-Moreno et al., 2019). Previous studies have shown that in harsh environments, plant resources are less available, and trait correlation intensifies. Spring ephemeral plants have a shorter lifespan and a lower temperatures environment during the growing season than spring non-ephemeral plants. Therefore, spring ephemeral plants exhibit a higher edge density and tighter trait correlations. Additionally, defensive traits were more prominent in the plant trait network of spring ephemeral plants, which also proved that spring ephemeral plants have a stronger adaptive capacity to changing environments. Thus, spring ephemeral plants employ cost-effective strategies, closely correlating traits to promote efficient functioning and greater adaptability to harsh environments.

The central traits of trait networks varied at different elevations. At low elevation, RNSC and LS are important central traits. Previous studies have shown that perennial plants increase their concentration of nonstructural carbohydrates to provide energy during the growing season. As elevation increased, LC and LP became key traits at middle and high elevations. Leaf carbon concentration (LC) reflects the energy cost of the leaf structure. Studies have shown that a high leaf carbon content results in high biomass cost per unit leaf area, high resistance to herbivorous attack, and long leaf longevity (Li et al., 2023). Phosphorus is crucial for nucleic acid and ATP synthesis, protein synthesis, and enhancing drought and cold resistance in plants (Chen et al., 2021). Moreover, we found that trait correlations were tighter at high and middle elevations, possibly due to low temperatures influencing plant resource availability (Liu et al., 2019). Our results provide further evidence that trait correlations are influenced by resource availability. Meanwhile, the modularity of plant trait networks increased at middle and high elevations compared to low elevations. The independence of modules provides more possibilities for combinations of plant traits and promotes the diversification of plant strategies to adapt to environmental changes (Aquilué et al., 2020).

In this study, the 26 functional traits were categorized into economic, nutritional, and defensive traits. Wang et al. (2023) demonstrated that the importance of traits with different characteristics is related to the plant’s life form. In the plant trait network, defensive traits of spring ephemeral plants were more important than other traits. Defensive traits are influenced by abiotic factors such as low temperatures, precipitation, and soil nutrients. Spring ephemeral plants prioritize tolerance to adapt to harsh environments, investing in LTPC, RTPC, and RCC to strengthen structural and chemical defenses. Defensive traits are also influenced by biotic factors, including pathogens and herbivores. Compared to non-early spring plants, spring-flowering plants face lower pathogen pressures. In contrast, non-early spring plants exhibit stronger defenses against biotic stress, with significant induction of resistance enzymes, while spring plants show minimal enzyme expression during this period (Heil and Ploss, 2006). Therefore, compared to spring non-ephemeral plants, spring ephemeral plants prioritize linking defense and nutrient traits to improve their ability to defend against low temperatures and enhance nutrient accumulation for flowering and reproduction. Both economic and nutrient traits are more important than defense traits at different elevations and in the trait networks of spring non-ephemeral plants. Investment in economic traits can improve carbon and nitrogen storage efficiency and optimize resource allocation (Wright et al., 2004).

## 5 Conclusion

This study determined the variation in 26 plant functional traits between two plant types and along the elevation gradient. These variations elucidate the growth-defense trade-off strategies in spring ephemeral and non-ephemeral plants. We found that plant type explained most of the variation in traits. And the functional traits in this study were more strongly influenced by environmental factors than by phylogenetic constraints. Spring ephemeral plants showed higher carbon and nitrogen content and lower carbohydrate content compared to non-ephemeral plants, adopting a fast-growing resource competition strategy. With increasing elevation, plants showed higher LDMC, LTFC, and lower N content, tending to adopt a conservative strategy. The plant trait network showed higher modularity to adapt to harsh environments at higher elevations. The trait network of spring ephemeral plants exhibited higher modularity and edge density, with stronger linkages among traits and prioritized defensive trait linkages, suggesting greater resilience to stress. In conclusion, our results offer new insights into nutrient utilization and adaptation strategies of spring ephemeral plants based on economic, nutritional, and defensive traits and their networks, providing a theoretical basis for guiding the cultivation and breeding of early spring plants.

## Data Availability

The raw data supporting the conclusions of this article will be made available by the authors, without undue reservation.
