# Supplementary material for: Trade-off strategies between growth and defense of spring ephemeral plants in early spring
Source: Front Plant Sci. 2025 Jan 28;16:1503169. doi: 10.3389/fpls.2025.1503169 (PMC11810948; doi:10.3389/fpls.2025.1503169)
Supplement: Supplementary file 1 [file Table1.docx]

**Supporting Information**

**Trade-off strategies between growth and defense of spring ephemeral plants in early spring**

**Appendix S1**

Measurement of functional traits

**Appendix S2**

**Table S1** Species of important values of herb layer ranked in the top 15 in different elevations

**Table S2** The list of plant species in the study

**Table S3** List of plant traits and their categories, units and abbreviations

**Table S4** Key parameters of plant trait networks

**Table S5** Variance contribution of each trait

**Figure S1** Four spring ephemeral plants and four spring non-ephemeral plants.

**Figure S2** Effect of MAT on SLA, LN, RP, RC/N, RS, LTPC, RCC, RTPC of plants with different species.

**Figure S3** Effect of soil_C on LDMC, SLA, LN, LP, LC/N, RN/P, LTPC, LTFC, RCC, RTPC (a-j) of plants with different species.

**Figure S4** Effect of soil_N on LC, LC/N, LS, RS, RNSC, LCC (a-j) of plants with different species.

**Fig S5** Effect of soil_N on LDMC, SLA, LN, RP, RN/P, RS, LTPC, RCC, RTPC (a-i) of plants with different species.

**Appendix S1**

***Measurement of functional traits***

***Phosphorus***

The phosphorus (P) content was determined using a UV spectrophotometer after digestion with H_2_SO_4_ and H_2_O_2_. The dried powder (0.25 g) of the sample was digestion with H_2_SO_4_ and H_2_O_2_. The mixture was diluted to 50 mL by deionized water and 1.0 mL of 10% ascorbic acid solution was added and reacted for 30 s. Then the mixture was continued to be added 2.0 mL molybdate solution and the absorbance value was measured at 700 nm after 15 min.

***Soluble sugar and starch***

Soluble sugar and starch concentrations were determined by the anthrone method. Soluble sugars were first extracted twice from 0.1 g of powder using 2 mL of 80% ethanol. The residue was decomposed with 2 mL of 9.2 mol/L HClO_4_ and 4 mL of distilled water was added for starch determination. The concentrations of soluble sugars and starch were measured by absorbance at 625 nm using a UV visible spectrophotometer. Sugar concentration was calculated from the regression equation of glucose standard solution and starch concentration. The sum of soluble sugars and starch is referred to as total nonstructural carbohydrates.

***Total phenolic***

The dried powder (0.2 g) of the sample was mixed with 10 mL of 70% methanol for 15 min and centrifuged at 8000 rpm for 10 min at 4 °C. The supernatant was collected and the same procedure was repeated twice. Total phenol and flavonoid contents were determined from the methanolic extracts. The reaction mixture contained 5 mL of 10% Folin-Ciocalteu reagent, 4 mL of 7.5% sodium carbonate solution, 2.5 mL deionized water and 1 mL of the methanolic extract. The reaction mixture was incubated in the dark for 1 hour to develop color and the absorbance was determined at 760 nm. The standard curve was prepared using gallic acid.

***Total flavonoids***

The total flavonoid content was determined by the aluminum chloride reaction. The extracts of total flavonoids were consistent with total phenols. To the supernatant (0.5 mL), 0.15 mL of 5% NaNO_2_ solution was added and incubated for 5 min, followed by 0.15 mL of 10% AlCl_3_-6H_2_O solution. After 5 min, 1 mL of 1 M NaOH solution were added to the reaction mixture. The above reactions were carried out at room temperature. The absorbance was measured at 415 nm and a standard curve was constructed using rutin standards.

***Cellulose***

The dried powder (0.2 g) of the sample was mixed with 60 mL 60% H_2_SO_4_ and digest for 30 minutes. Dilute to 100 mL with 60% H_2_SO_4_ and filter with a Buchner funnel. Take 2 mL of supernatant and add 0.5 mL of 2% anthrone reagent, and add 5 mL of concentrated H_2_SO_4_, let it stand for 12 min, then measure the absorbance at 620 nm.

***Lignin***

The dried powder (0.5 g) of the sample was mixed with 50 mL 2% NaOH then heated in an 80 ℃ water bath for 2 h. After filtration, 1 mL11% HCI solution, 10 mL of ethanol, 1 mL of phenol, and then 10 mL of ethanol were added to the filtrate sequentially. Finally, the filtrate was fixed to 100 mL with deionized water. The absorbance of the mixture was determined at 280 nm.

**Appendix S2**

**Table S1** Species of important values of herb layer ranked in the top 15 in different elevations

| **Site** | **Species** | **Relative density (%)** | **Relative** **coverage (%)** | **Relative frequency (%)** | **Important value**  **(%)** |
| --- | --- | --- | --- | --- | --- |
| L | *Meehania urticifolia* (Miq.) Makino | 0.48 | 0.40 | 0.19 | 35.59 |
|  | *Filipendula palmata* (Pall.) Maxim. | 0.09 | 0.11 | 0.11 | 10.22 |
|  | ***Angelica dahurica* (Fisch. ex Hoffm.) Benth. et Hook. f. ex Franch. et Sav.** | 0.12 | 0.10 | 0.07 | 9.67 |
|  | ***Aegopodium alpestre*Ledeb.** | 0.08 | 0.08 | 0.04 | 6.72 |
|  | ***Anemone raddeana* Regel** | 0.08 | 0.08 | 0.04 | 6.72 |
|  | ***Erythronium japonicum* Decne.** | 0.03 | 0.05 | 0.04 | 3.83 |
|  | ***Adonis amurensis* Regel et Radde** | 0.006 | 0.01 | 0.07 | 3.09 |
|  | ***Hylomecon japonica* (Thunb.) Prantl** | 0.02 | 0.03 | 0.04 | 2.83 |
|  | ***Cardamine leucantha*(Tausch) O. E. Schulz** | 0.02 | 0.02 | 0.04 | 2.58 |
|  | *Lilium dauricum* Ker Gawl. | 0.02 | 0.02 | 0.04 | 2.55 |
|  | *Lilium distichum* Nakai ex Kamibayashi | 0.01 | 0.02 | 0.04 | 2.37 |
|  | *Saussurea ovata* Benth. | 0.01 | 0.02 | 0.04 | 2.23 |
|  | *Asarum heterotropoides* F. Schmidt var. *mandshuricum* (Maxim.) Kitag. | 0.006 | 0.01 | 0.04 | 1.86 |
|  | ***Pimpinella brachycarpa* (Kom.) Nakai** | 0.003 | 0.01 | 0.04 | 1.75 |
|  | *Rubia cordifolia* L. | 0.006 | 0.008 | 0.04 | 1.72 |
| M | ***Hylomecon japonica* (Thunb.) Prantl** | 0.15 | 0.20 | 0.09 | 14.79 |
|  | *Anemone amurensis* (Korsh.) Kom. | 0.20 | 0.11 | 0.11 | 13.96 |
|  | ***Anemone raddeana* Regel** | 0.13 | 0.09 | 0.07 | 9.62 |
|  | *Maianthemum japonicum* (A. Gray) LaFrankie | 0.07 | 0.09 | 0.09 | 8.38 |
|  | ***Cardamine leucantha*(Tausch) O. E. Schulz** | 0.10 | 0.06 | 0.09 | 8.23 |
|  | *Corydalis fumariifolia* Maxim. | 0.07 | 0.09 | 0.07 | 7.55 |
|  | ***Angelica dahurica* (Fisch. ex Hoffm.) Benth. et Hook. f. ex Franch. et Sav.** | 0.09 | 0.07 | 0.07 | 7.36 |
|  | *Ranunculus franchetii* H. Boissieu | 0.03 | 0.03 | 0.09 | 5.12 |
|  | *Paris verticillata* M. Bieb. | 0.05 | 0.05 | 0.04 | 4.53 |
|  | ***Erythronium japonicum* Decne.** | 0.02 | 0.06 | 0.04 | 4.07 |
|  | *Arisaema amurense* Maxim. | 0.02 | 0.02 | 0.07 | 3.43 |
|  | ***Adonis amurensis* Regel et Radde** | 0.02 | 0.05 | 0.02 | 3.09 |
|  | *Corydalis ussuriensis* Aparina | 0.01 | 0.02 | 0.04 | 2.17 |
|  | ***Pimpinella brachycarpa* (Kom.) Nakai** | 0.01 | 0.02 | 0.02 | 1.76 |
|  | ***Aegopodium alpestre*Ledeb.** | 0.011 | 0.01 | 0.02 | 1.48 |
| H | ***Erythronium japonicum* Decne.** | 0.32 | 0.38 | 0.13 | 27.46 |
|  | *Gymnospermium microrrhynchum* (S. Moore) Takht. | 0.14 | 0.14 | 0.05 | 10.97 |
|  | ***Anemone raddeana* Regel** | 0.14 | 0.05 | 0.10 | 9.88 |
|  | *Maianthemum japonicum* (A. Gray) LaFrankie | 0.09 | 0.10 | 0.10 | 9.62 |
|  | ***Hylomecon japonica* (Thunb.) Prantl** | 0.05 | 0.05 | 0.10 | 6.86 |
|  | *Aconitum kusnezoffii* Reichb. var. *gibbiferum* (Reichb.) Regel | 0.05 | 0.07 | 0.05 | 5.52 |
|  | *Meehania urticifolia* (Miq.) Makino | 0.06 | 0.05 | 0.05 | 5.33 |
|  | ***Aegopodium alpestre* Ledeb.** | 0.05 | 0.04 | 0.03 | 3.84 |
|  | *Anemone amurensis* (Korsh.) Kom. | 0.03 | 0.02 | 0.05 | 3.59 |
|  | ***Adonis amurensis* Regel et Radde** | 0.01 | 0.01 | 0.05 | 2.47 |
|  | ***Cardamine leucantha*(Tausch) O. E. Schulz** | 0.01 | 0.01 | 0.05 | 2.35 |
|  | *Asarum sieboldii* Miq. f. *seoulense* (Nakai) C. Y. Cheng et C. S. Yang | 0.007 | 0.01 | 0.05 | 2.29 |
|  | ***Pimpinella brachycarpa* (Kom.) Nakai** | 0.02 | 0.02 | 0.03 | 2.09 |
|  | ***Angelica dahurica* (Fisch. ex Hoffm.) Benth. et Hook. f. ex Franch. et Sav.** | 0.01 | 0.01 | 0.03 | 1.49 |
|  | *Isopyrum manshuricum* Kom. | 0.01 | 0.01 | 0.03 | 1.45 |

**Note:** Bolded species indicate that they occur at every elevation.

**Table S2** The list of plant species in the study

| **Categories** | **Species** | **Genus** | **Family** |
| --- | --- | --- | --- |
| Spring ephemerals plants | *Anemone raddeana*Regel | Anemone | Ranunculaceae |
|  | *Erythronium japonicum* Decne. | Erythronium | Liliaceae |
|  | *Adonis amurensis* Regel & Radde | Adonis | Ranunculaceae |
|  | *Hylomecon Japonicum* (Thunb.) Prantl | Hylomecon | Papaveraceae |
| Spring emerging plants | *Angelica dahurica* (Fisch. ex Hoffm.) Benth. & Hook. f. ex Franch. & Sav. | Angelica | Apiaceae |
|  | *Aegopodium alpestre* Ledeb. | Aegopodium | Apiaceae |
|  | *Cardamine leucantha* (Tausch) O. E. Schulz | Cardamine | Brassicaceae |
|  | *Pimpinella brachycarpa* (Kom.) Nakai | Pimpinella | Apiaceae |

**Table S3** List of plant traits and their categories, units and abbreviations

| **Categories** | **Abbreviation** | **Variables** | **Units** | **References** |
| --- | --- | --- | --- | --- |
| Economic traits | LDMC | Leaf dry matter content | % | (Li *et al.* 2022) |
|  | SLA | Specific leaf area | cm^2^ g^-1^ | (Li *et al.* 2022) |
|  | LC* | Leaf carbon concentration | % | (Li *et al.* 2022) |
|  | LN* | Leaf nitrogen concentration | % | (Li *et al.* 2022) |
|  | LP* | Leaf phosphorus concentration | mg g^-1^ | (Li *et al.* 2022) |
|  | LC/N* | The ratio of carbon to nitrogen concentration of leaf | - | (Li *et al.* 2022) |
|  | LN/P* | The ratio of nitrogen to phosphorus concentration of leaf | - | (Li *et al.* 2022) |
| nutrient­ traits | RC | Root carbon concentration | % | (Li *et al.* 2022) |
|  | RN | Root nitrogen concentration | % | (Li *et al.* 2022) |
|  | RP | Root phosphorus concentration | mg g^-1^ | (Li *et al.* 2022) |
|  | RC/N | The ratio of carbon to nitrogen concentration of root | - | (Li *et al.* 2022) |
|  | RN/P | The ratio of nitrogen and phosphorus concentration of root | - | (Li *et al.* 2022) |
|  | LSS | Leaf soluble sugar concentration | mg g^-1^ | (Rao *et al.* 2021) |
|  | LS | Leaf starch concentration | mg g^-1^ | (Rao *et al.* 2021) |
|  | LNSC | Leaf nonstructural carbohydrates | mg g^-1^ | (Rao *et al.* 2021) |
|  | RSS | Root soluble sugar concentration | mg g^-1^ | (Rao *et al.* 2021) |
|  | RS | Root starch concentration | mg g^-1^ | (Rao *et al.* 2021) |
|  | RNSC | Root nonstructural carbohydrates | mg g^-1^ | (Rao *et al.* 2021) |
| Defensive traits | LCC | Leaf cellulose content | % | (Wang *et al.* 2022) |
|  | LLC | Leaf lignin content | % | (Wang *et al.* 2022) |
|  | LTPC | Leaf total phenols concentration | mg g^-1^ | (Wang *et al.* 2022) |
|  | LTFC | Leaf total flavonoids concentration | mg g^-1^ | (Wang *et al.* 2022) |
|  | RCC | Root cellulose content | % | (Wang *et al.* 2022) |
|  | RLC | Root lignin content | % | (Wang *et al.* 2022) |
|  | RTPC | Root total phenols concentration | mg g^-1^ | (Wang *et al.* 2022) |
|  | RTFC | Root total flavonoids concentration | mg g^-1^ | (Wang *et al.* 2022) |

**Note**: * indicates that the trait is both an economic and a nutrient trait.

**Table S4** Key parameters of plant trait networks

| **Categories** | **Parameters** | **Definition** | **Ecological significance** |
| --- | --- | --- | --- |
| Node parameter | Degree (K) | K was the number of edges that connect a focal node trait to other nodes. | Plant traits with high k values can be considered as overall hub traits, which are beneficial for the effective utilization and acquisition of resources within and between plant tissues. |
| Overall parameters | Edge density (ED) | ED was defined as the density of the connected edges between nodes in a network, that is, the proportion of actual connections among traits out of all possible connections. | Networks with higher ED allow for more efficient access and mobilization of resources. |
|  | Modularity (M) | M was defined as clusters of traits that exhibit covariation among themselves, relatively independently of other  clusters. | Plant trait networks with higher M values are more tightly linked within the modules and more loosely linked externally which confers an advantage under variable conditions. |

**Table S5** Variance contribution of each trait

| **Variables** | **PC1** | **PC2** |
| --- | --- | --- |
| Leaf starch concentration (LS) | **0.30666** | 0.01606 |
| Leaf carbon concentration (LC) | **0.28919** | 0.05652 |
| Leaf phosphorus concentration (LP) | **0.28528** | 0.02447 |
| The ratio of nitrogen to phosphorus concentration of leaf (LN/P) | **0.28104** | 0.08068 |
| Root starch sugar concentration (RS) | **0.27706** | 0.01646 |
| Leaf total phenols concentration (LTPC) | 0.27259 | 0.16361 |
| Root phosphorus concentration (RP) | 0.2583 | 0.04036 |
| Specific leaf area (SLA) | 0.25826 | 0.2384 |
| The ratio of carbon to nitrogen concentration of root (RC/N) | 0.24263 | 0.17371 |
| Root nonstructural carbohydrates (RNSC) | 0.23683 | 0.03163 |
| Root carbon concentration (RC) | 0.23314 | 0.00476 |
| Root nitrogen concentration (RN) | 0.20509 | 0.20592 |
| Leaf nitrogen concentration (LN) | 0.20324 | 0.14775 |
| Leaf nonstructural carbohydrates (LNSC) | 0.19844 | **0.3068** |
| The ratio of nitrogen and phosphorus concentration of root (RN/P) | 0.17336 | 0.08265 |
| Root cellulose concentration (RCC) | 0.11923 | 0.27213 |
| Root total phenols concentration (RTPC) | 0.11056 | **0.31141** |
| Leaf lignin content (LLC) | 0.09525 | 0.07743 |
| The ratio of carbon to nitrogen concentration of leaf (LC/N) | 0.0938 | 0.15783 |
| Root soluble sugar concentration (RSS) | 0.07543 | 0.09099 |
| Leaf soluble sugar concentration (LSS) | 0.05115 | **0.39491** |
| Leaf total flavonoids concentration (LTFC) | 0.04588 | 0.26968 |
| Leaf dry matter content (LDMC) | 0.01985 | 0.00025 |
| Leaf cellulose concentration (LCC) | 0.01315 | **0.36637** |
| Root lignin content (RLC) | 0.00695 | 0.22372 |
| Root total flavonoids concentration (RTFC) | 0.00515 | **0.29046** |

**Note:** bolded text indicates top 5 contributions in PC1 and PC2.


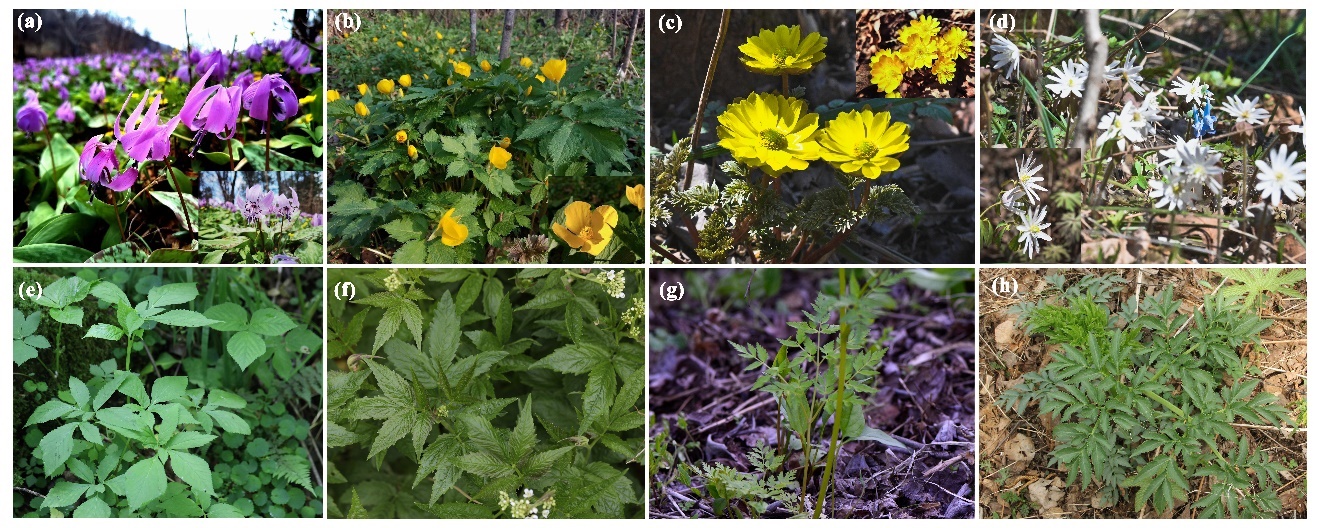


**Figure S1** Four spring ephemeral plants and four spring non-ephemeral plants. (a) *Erythronium japonicum* Decne.; (b) *Hylomecon Japonicum* (Thunb.) Prantl; (c) *Adonis amurensis* Regel & Radde; (d) *Anemone raddeana* Regel; (e) *Pimpinella brachycarpa* (Kom.) Nakai; (f) *Cardamine leucantha* (Tausch) O. E. Schulz; (g) *Aegopodium alpestre* Ledeb.; (h) *Angelica dahurica* (Fisch. ex Hoffm.) Benth. & Hook. f. ex Franch. & Sav.


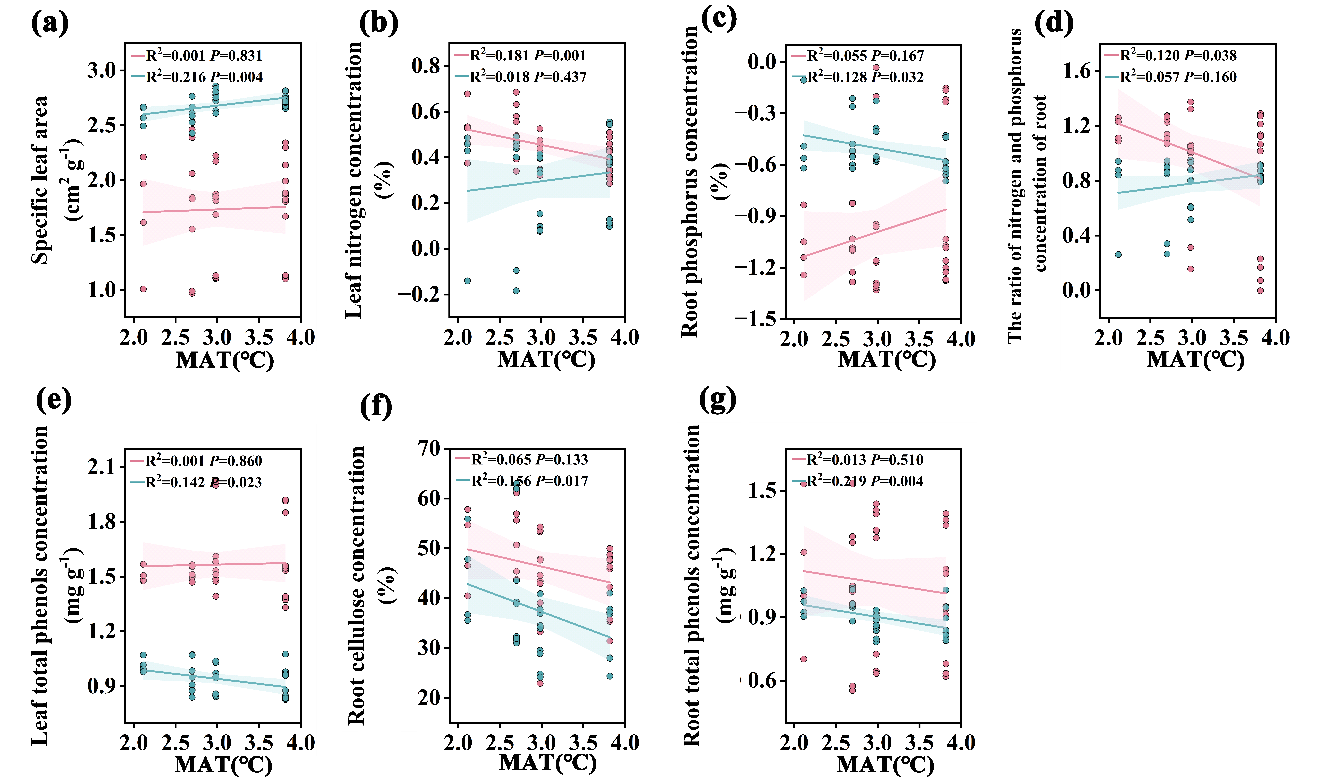


**Figure S2** Effect of MAT on SLA, LN, RP, RC/N, RS, LTPC, RCC, RTPC (a-g) of plants with different species. Spring ephemeral plants were represented by pink and spring non-ephemeral plants were represented by blue. The shaded area around the line represents the 95% confidence interval of the regression. Abbreviations: specific leaf area (SLA), leaf nitrogen concentration (LN), root phosphorus concentration (RP), the ratio of carbon to nitrogen concentration of root (RC/N), root starch concentration (RS), leaf total phenols concentration (LTPC), root cellulose content (RCC), and Root total phenols concentration (RTPC).


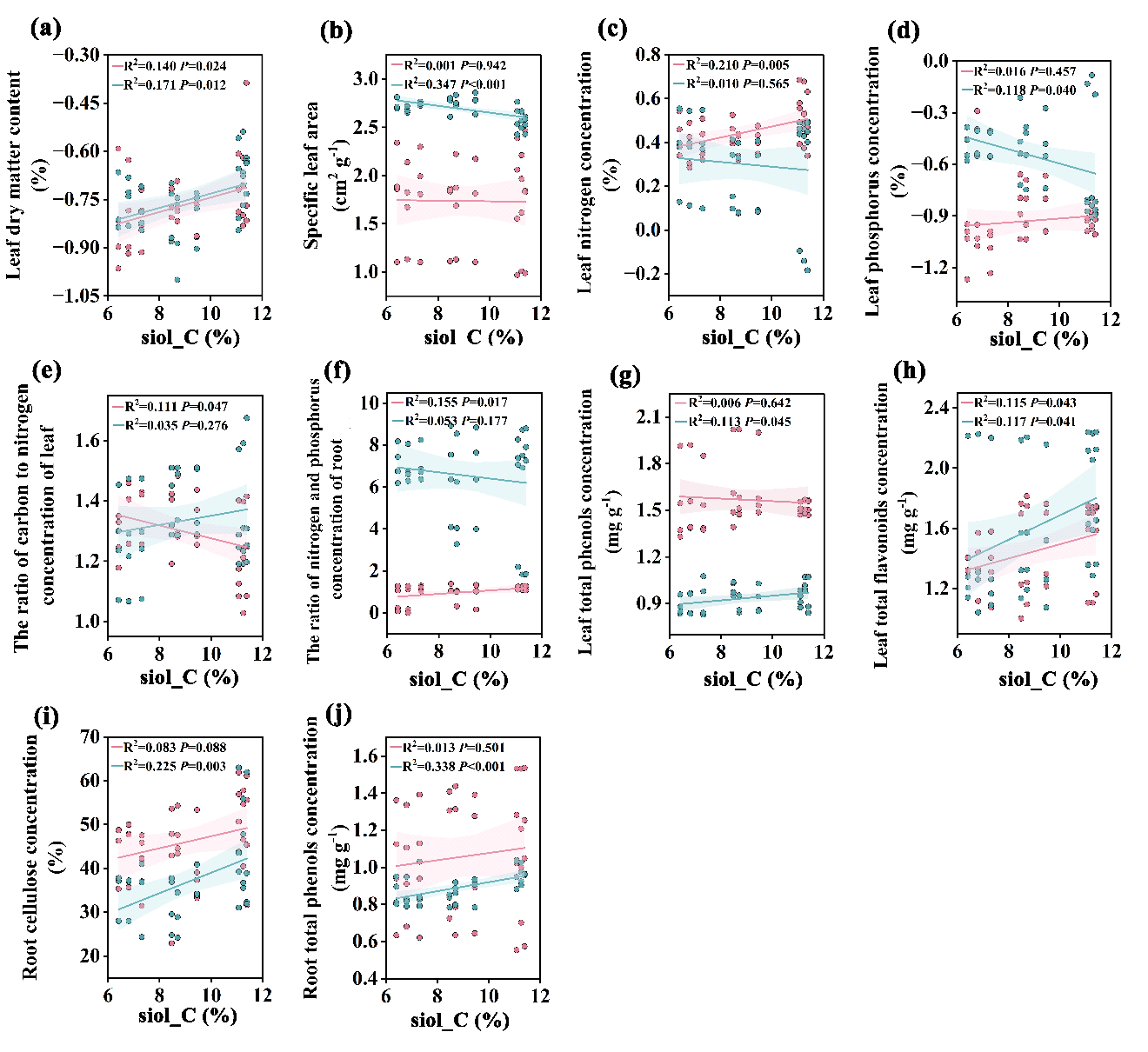


**Figure S3** Effect of soil_C on LDMC, SLA, LN, LP, LC/N, RN/P, LTPC, LTFC, RCC, RTPC (a-j) of plants with different species. Spring ephemeral plants were represented by pink and spring non-ephemeral plants were represented by blue. The shaded area around the line represents the 95% confidence interval of the regression. Abbreviations: leaf dry matter content (LDMC), specific leaf area (SLA), leaf nitrogen concentration (LN),

leaf phosphorus concentration (LP), the ratio of carbon to nitrogen concentration of leaf (LC/N), the ratio of nitrogen and phosphorus concentration of root (RN/P), leaf total phenols concentration (LTPC), leaf total flavonoids concentration (LTFC), root cellulose content (RCC), and root total phenols concentration (RTPC).


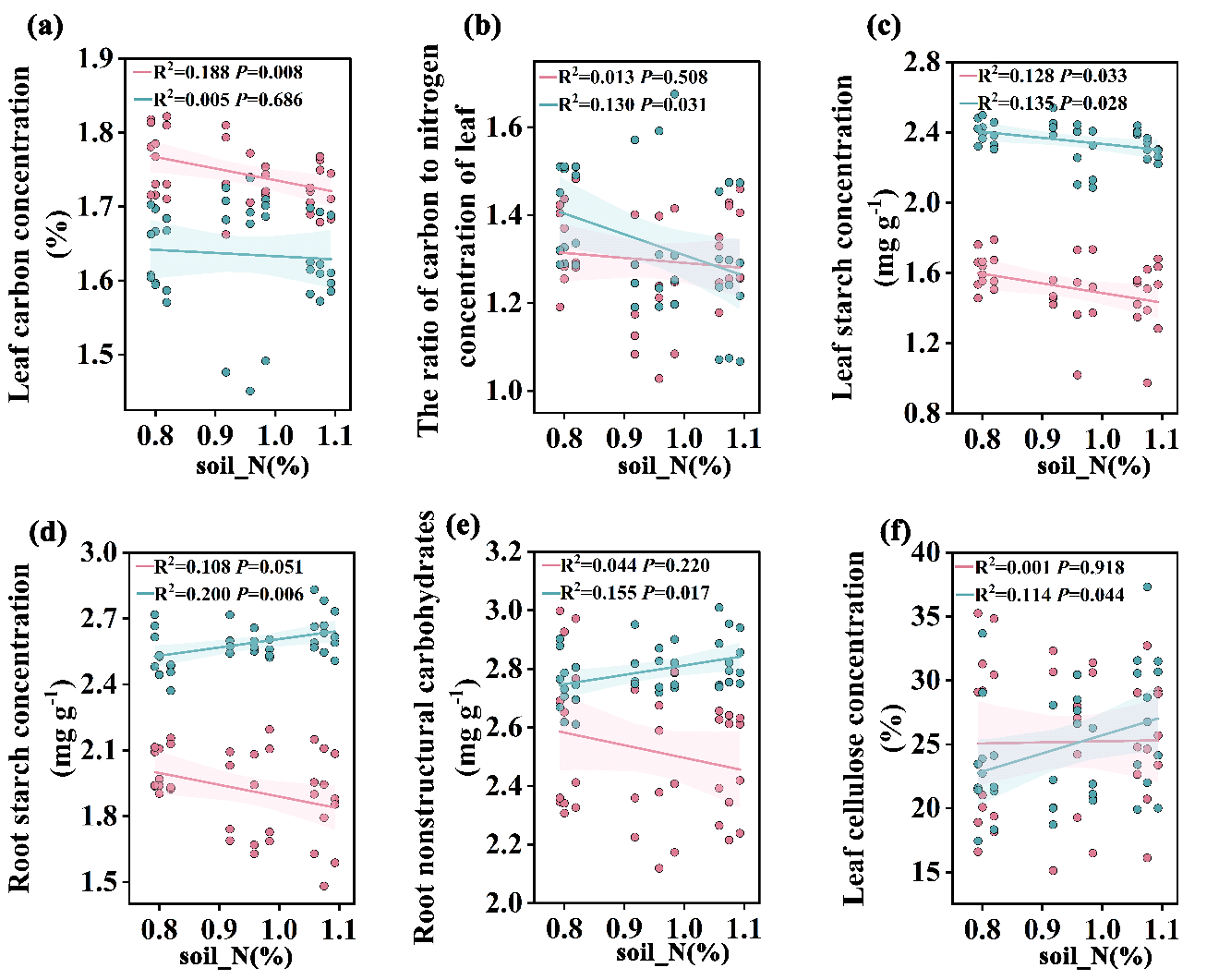


**Figure S4** Effect of soil_N on LC, LC/N, LS, RS, RNSC, LCC (a-j) of plants with different species. Spring ephemeral plants were represented by pink and spring non-ephemeral plants were represented by blue. The shaded area around the line represents the 95% confidence interval of the regression. Abbreviations: leaf carbon concentration (LC), the ratio of carbon to nitrogen concentration of leaf (LC/N), leaf starch concentration (LS), root starch concentration (RS), root nonstructural carbohydrates (RNSC), leaf cellulose concentration (LCC).


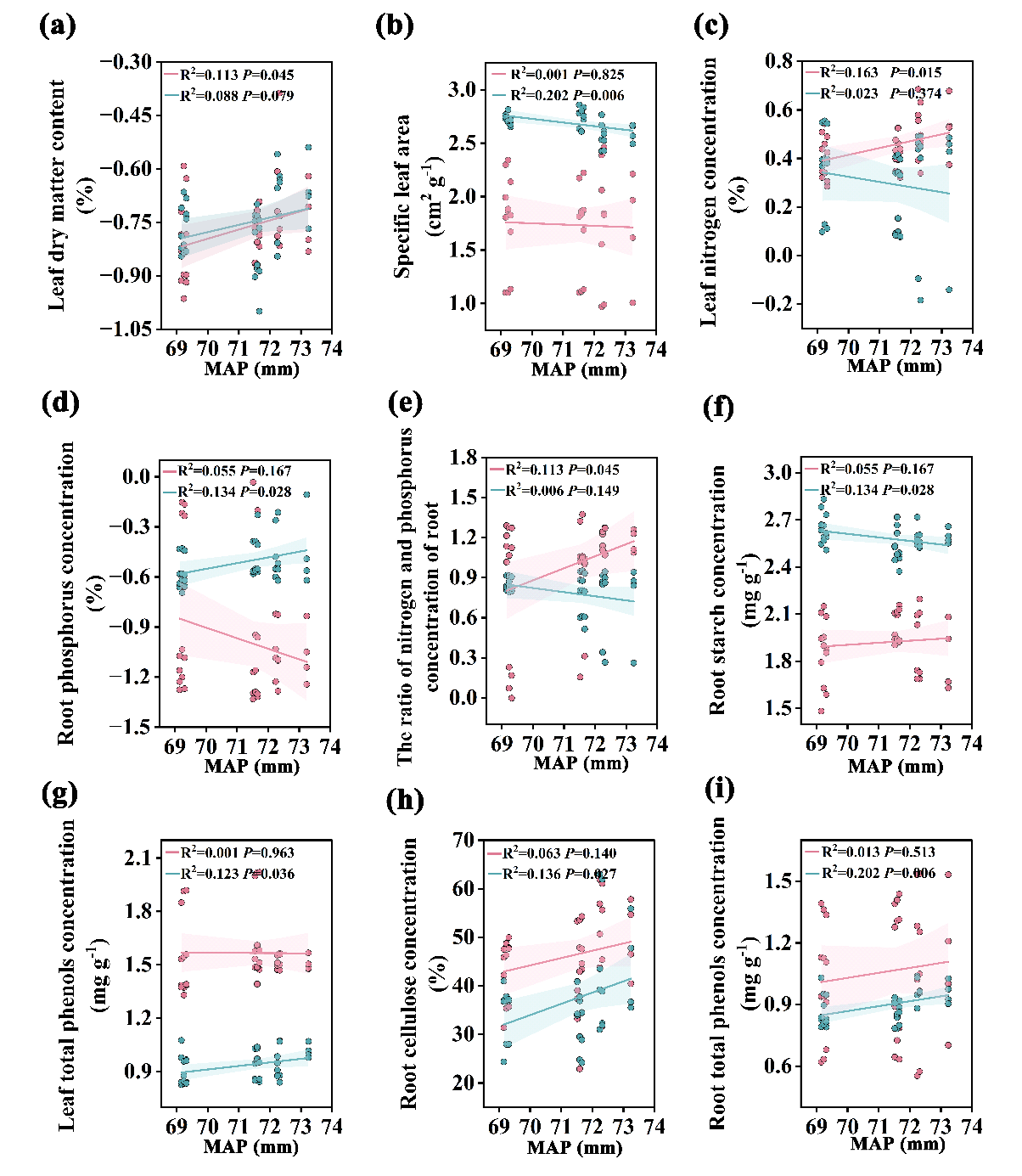


**Figure S5** Effect of MAP on LDMC, SLA, LN, RP, RN/P, RS, LTPC, RCC, RTPC (a-i) of plants with different species. Spring ephemeral plants were represented by pink and spring non-ephemeral plants were represented by blue. The shaded area around the line represents the 95% confidence interval of the regression. Abbreviations: leaf dry matter content (LDMC), specific leaf area (SLA), leaf nitrogen concentration (LN), root phosphorus concentration (RP), the ratio of nitrogen and phosphorus concentration of root (RN/P), root starch concentration (RS), leaf total phenols concentration (LTPC), root cellulose content (RCC), and root total phenols concentration (RTPC).

**References**

1.

Li, Y., Liu, C.C., Sack, L., Xu, L., Li, M.X., Zhang, J.H. *et al.* (2022). Leaf trait network architecture shifts with species-richness and climate across forests at continental scale. *Ecol Lett*, 25, 1442-1457.

2.

Rao, Q.Y., Su, H.J., Ruan, L.W., Deng, X.W., Wang, L.N., Rao, X. *et al.* (2021). Stoichiometric and physiological mechanisms that link hub traits of submerged macrophytes with ecosystem structure and functioning. *Water Res*, 202.

3.

Wang, X.Z., Sun, S.W., Sedio, B.E., Glomglieng, S., Cao, M., Cao, K.F. *et al.* (2022). Niche differentiation along multiple functional-trait dimensions contributes to high local diversity of Euphorbiaceae in a tropical tree assemblage. *J Ecol*, 110, 2731-2744.
